# Early, very high-dose, and prolonged vitamin C administration in murine sepsis

**DOI:** 10.1038/s41598-025-02622-7

**Published:** 2025-05-20

**Authors:** Ok-Hyeon Kim, Tae Wan Kim, Hana Kang, Tae Jin Jeon, Eun Seo Chang, Hyun Jung Lee, Won-Young Kim

**Affiliations:** 1 Department of Anatomy and Cell Biology, Chung-Ang University College of Medicine, Seoul, Republic of Korea; 2 Division of Pulmonary and Critical Care Medicine, Department of Internal Medicine, Chung-Ang University Hospital, Chung-Ang University College of Medicine, Seoul, Republic of Korea; 3 Department of Global Innovative Drugs, Graduate School of Chung-Ang University, Seoul, Republic of Korea

## Abstract

This proof-of-concept study aimed to assess the optimal timing, dosing, and duration of vitamin C administration to increase survival and attenuate organ injuries in murine sepsis. Mice were randomized to receive ascorbic acid (AscA) at 1 or 6 h after cecal ligation and puncture (CLP). At each time point, mice randomly received AscA for 4 or 8 d. Mice were assigned to sham and CLP groups, as well as CLP + AscA groups that were treated with AscA at doses of 90, 180, or 360 mg/kg/d. The survival curves diverged significantly when AscA was injected at doses of 180 or 360 mg/kg/d for 8 d, although this was not observed when the treatment was limited to 4 d. AscA at doses of 180 or 360 mg/kg/d for 8 d preserved lung architecture while attenuating the abnormal expression of tight junction proteins. Kidney and liver injuries were evident in CLP mice, with elevated expression of biomarkers and inflammatory mediators; however, exposure to AscA at doses of 180 or 360 mg/kg/d for 8 d improved the histological changes and decreased biomarker expression levels. Very high-dose and prolonged vitamin C administration may potentially play a role in the management of sepsis-associated organ injuries.

**Supplementary Information:**

The online version contains supplementary material available at 10.1038/s41598-025-02622-7.

## Introduction

Sepsis results from a dysregulated host response to infection and is often accompanied by persistent inflammation, organ dysfunction, and death^1^. Despite improvements in sepsis management over the last 30 years, sepsis-related mortality remains high, with 11 million deaths occurring each year globally^2^. Vitamin C has been suggested as a treatment option for sepsis and septic shock due to its anti-inflammatory and antioxidant properties^3^. To investigate its potential effects in this population, numerous randomized trials of vitamin C as monotherapy and in combination with hydrocortisone and thiamine have been conducted^4^. Although modest improvements in vasopressor dose and organ function scores were observed, most trials did not demonstrate a survival benefit.

Despite these numerous studies, questions regarding the optimal timing, dosing, and duration of vitamin C therapy remain unanswered. First, early use of vitamin C was not assessed in previous studies, which may have contributed to ineffective outcomes^5^. Second, clinical uncertainty persists for optimal dosing, as a recent meta-analysis revealed the association between a very high-dose (≥ 12 g/d) of vitamin C and decreased mortality^6^. Third, patients may develop hypovitaminosis within 48 h after discontinuing vitamin C infusion, regardless of the dosing regimen^7^. However, the 4-d treatment period was selected arbitrarily in previous trials, which may not translate to an improvement in mortality. Altogether, the dosing regimen used in previous studies may have been “too late, too little, and too short” to observe any treatment effects, although these factors require further investigation.

To address these controversial issues, this study investigated whether early, very high-dose, and/or prolonged vitamin C treatment could increase survival and attenuate multiple organ injury in a well-characterized murine model of sepsis.

## Methods

### Experimental protocol

Female C57BL/6 mice (10–12-week-old, 18–20 g) were purchased from DBL (Eumseong-gun, Chungcheongbuk-do, Korea). All mice were housed in a cage, with a density of five mice per cage, and maintained with a normal laboratory diet and tap water ad libitum in an air-conditioned room (21 ± 2 °C) with a 12-h light–dark cycle. The cecal ligation and puncture (CLP) procedure was performed, as previously described^8,9^. Briefly, the mice were anesthetized with isoflurane (2–4% for induction and 1–3% for maintenance), and their abdomens were shaved and prepared with 70% ethanol. A midline laparotomy was performed, and the cecum ligated approximately 1 cm distal to the ileocecal valve and punctured using a 21-guage needle. The cecum was then gently squeezed to express a small amount of feces and returned to the abdominal cavity. The sham group underwent the same procedure but without CLP. The abdomen was closed in layers, and the mice injected subcutaneously with saline (1 mL) and imipenem (25 mg/kg; Sigma-Aldrich, St. Louis, MO, USA) for fluid resuscitation and infection prevention, respectively.

**Fig. 1:**
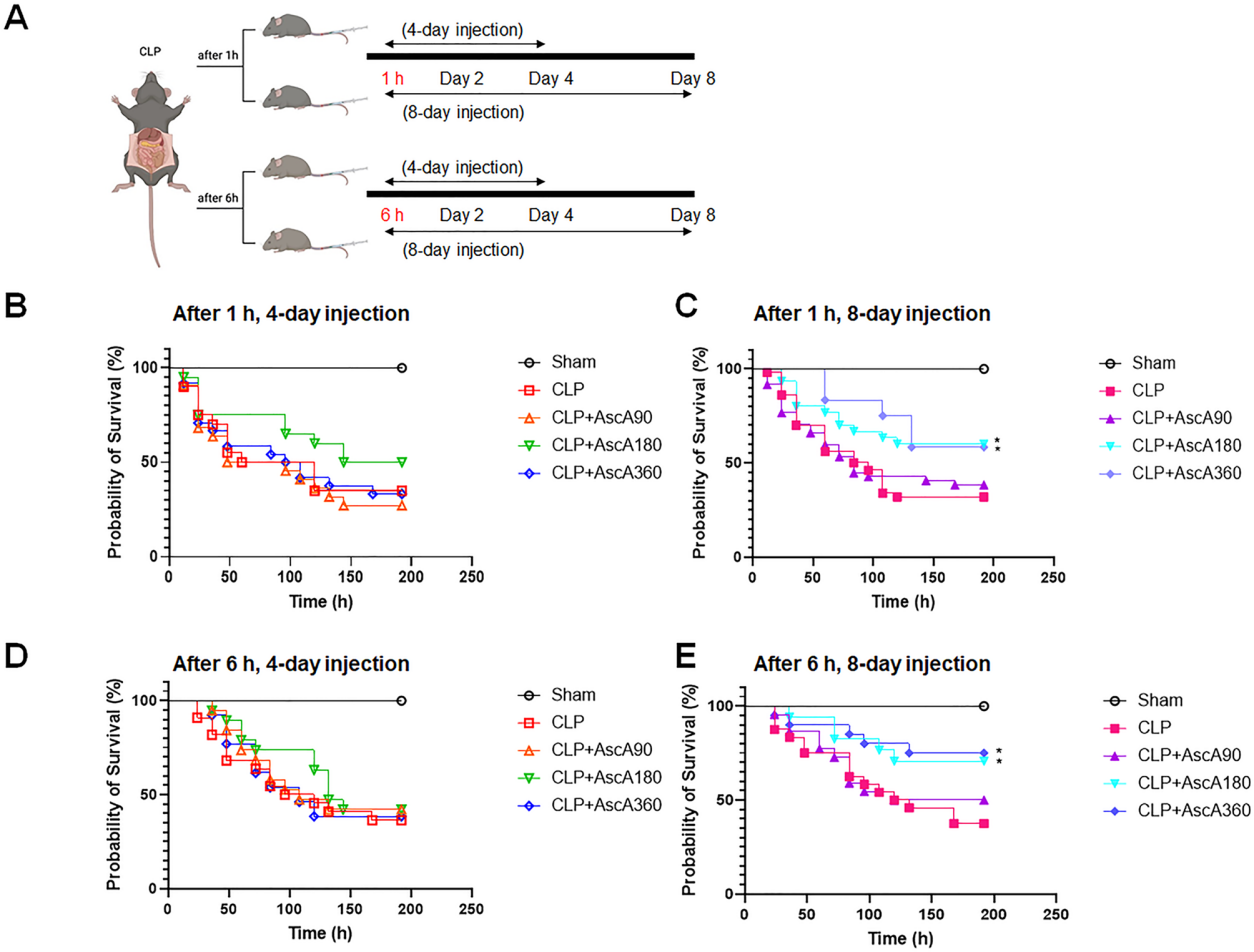
Effect of timing, dosing, and duration of vitamin C therapy on survival in septic mice. (**A**) Experimental design. (**B**) Ascorbic acid (AscA) was injected 1 h after cecal ligation and puncture (CLP) for 4 d (n = 9 in the sham; n = 20 in the CLP; n = 22 in the CLP + AscA90; n = 20 in the CLP + AscA180; and n = 24 in the CLP + AscA360). (**C**) AscA was injected 1 h after CLP for 8 d (n = 18 in the sham; n = 50 in the CLP; n = 47 in the CLP + AscA90; n = 30 in the CLP + AscA180; and n = 12 in the CLP + AscA360). (**D**) AscA was injected 6 h after CLP for 4 d (n = 9 in the sham; n = 22 in the CLP; n = 19 in the CLP + AscA90; n = 19 in the CLP + AscA180; and n = 13 in the CLP + AscA360). (**E**) AscA was injected 6 h after CLP for 8 d (n = 12 in the sham; n = 24 in the CLP; n = 22 in the CLP + AscA90; n = 17 in the CLP + AscA180; and n = 20 in the CLP + AscA360). *P < 0.05 when compared to the CLP group; log-rank test.

Vitamin C-treated mice received intravenous ascorbic acid (AscA) at doses of 90, 180, or 360 mg/kg/d in the lateral tail vein. Untreated mice received the vehicle (saline) instead of AscA. Mice were randomized into four groups: 1) those that received AscA 1 h after CLP and every 12 h for 4 d; 2) those that received AscA 1 h after CLP and every 12 h for 8 d; 3) those that received AscA 6 h after CLP and every 12 h for 4 d; and 4) those that received AscA 6 h after CLP and every 12 h for 8 d (Fig. 1A). The optimal cutoff values for initial timing of vitamin C administration were determined based on the survival rate and time-dependent interleukin (IL)-6 level (see Supplementary Fig. S1, S2). The duration of 8 d was based on a hypothesis that limiting the use of vitamin C to 4 d, as assessed in most previous studies^4^, may not lead to a survival benefit. The investigators were blinded to group allocation until data collection. Mice were euthanized 8 d after CLP induction, and the lung, kidney, and liver tissues were harvested for histological and biochemical analyses. For euthanasia procedures, animals were placed in a chamber filled with carbon dioxide at a displacement rate of 50% of the chamber volume per minute for 2 min. After exposure, the animals remained undisturbed in the chamber for additional 2 min. The experimental procedures were approved by the Institutional Animal Care and Use Committee of Chung-Ang University (Seoul, Korea; A2022023). All animal experiments were performed in accordance with the ARRIVE guidelines 2.0 (see Supplementary Appendix).

### Murine sepsis score

The murine sepsis score (MSS) was developed to predict disease progression and mortality in an animal model of polymicrobial sepsis^10^. The score consists of seven clinical variables: appearance; level of consciousness; activity; response to stimulus; eyes; respiration rate; and respiration quality (see Supplementary Table S1). Each of these variables was given a score between 0 and 4, and the MSS comprised the average of these seven scores.

### Measurement of proinflammatory cytokine levels

At 24 h after CLP, the distal tail was cut and peripheral blood drawn into a pipette tip. The samples were centrifuged for 15 min at 3000 rpm and 4 °C, and the serum was stored at − 80 °C for analysis. To determine the levels of proinflammatory cytokines, those of IL-6 were measured using enzyme-linked immunosorbent assay kits (AbFrontier, Seoul, Korea), according to the manufacturer’s instructions.

### Histological assessment of organ injury

Lung, kidney, and liver tissues were fixed in 10% neutral-buffered formalin (Biosesang, Seongnam-si, Gyeonggi-do, Korea), dehydrated, and embedded in paraffin blocks. Tissue sections of 4-µm thickness were obtained from the samples, which were then fixed onto a glass slide, dried, and stained. The slices were then soaked in xylene, gradient concentrations of ethanol, and hematoxylin–eosin. After mounting, according to the manufacturer’s instructions, the sections were dried, examined, and photographed using an inverted microscope equipped with a Leica camera (Leica Microsystem, Wetzlar, Hesse, Germany). The severity of lung injury was scored in a blinded manner as 0 (none or minimal), 1 (mild), 2 (moderate), or 3 (severe) based on the presence of lung edema, pulmonary congestion, thickening of the alveolar wall, and areas of inflammatory infiltration^11^. Kidney injury was determined by the percentage of dead area, defined as tubular dilatation, epithelial necrosis, and intratubular cast formation, and was defined as 0 (no damage), 1 (< 25%), 2 (25–50%), 3 (50–75%), and 4 (> 75%)^12^. To assess liver injury, an injury grading score was created based on the severity of necrotic lesions in the liver parenchyma^13^. The grades are as follows: 0 (no pathologic changes), 1 (presence of degenerative hepatocytes with only rare foci of necrosis), 2 (small areas of mild centrilobular necrosis around the central vein), 3 (areas of mild centrilobular necrosis more severe than grade 2), and 4 (centrilobular necrosis more severe than grade 3). The sum of the scores of each animal was averaged.

### RNA isolation and quantitative real-time PCR

Total RNA was extracted and purified from the lung, kidney, and liver tissues using TRIzol (Thermo Fisher Scientific, Waltham, MA, USA). RNA was reverse-transcribed using the Maxima First Strand cDNA Synthesis Kit (Thermo Fisher Scientific). Real-time PCR (RT-PCR) was performed using the Power SYBR Green PCR Master Mix in a StepOnePlus Real-Time PCR System (Applied Biosystems, Foster City, CA, USA). The primers used for RT-PCR are listed in Supplementary Table S2. Cycle thresholds were calculated using StepOne software v2.3 (Applied Biosystems), and mRNA levels were normalized to those of GAPDH.

### Western blotting analysis

Liver tissues were lysed in RIPA buffer (Thermo Fisher Scientific) containing 1% protease inhibitor cocktail and a phosphatase inhibitor cocktail (GenDEPOT, Katy, TX, USA). Total protein concentrations were determined using the Bradford Protein Assay Kit (Bio-Rad, Hercules, CA, USA), and equal amounts separated via 4–20% SDS-PAGE and analyzed through immunoblotting. Rabbit anti-cyclooxygenase-2 (ab102005; Abcam, Cambridge, UK), rabbit anti-heme oxygenase-1 (ab13243; Abcam), and anti-GAPDH (sc-365062; Santa Cruz Biotechnology, Dallas, TX, USA) were used for this analysis. The expression levels of housekeeping genes vary among animals. Thus, the expression of each target protein was normalized to its corresponding GAPDH level in the same individual, and quantitative graphs were generated accordingly (Fig. 5E). Gel images were captured with an ImageQuant LAS 4000 system (GE Healthcare Life Sciences, Chicago, IL, USA). Full unedited gel images for Fig. 5D are shown in Supplementary Fig. S3.

### Statistical analysis

All data are presented as the mean ± standard error of the mean. The results were compared using one-way analysis of variance and Tukey’s post-hoc test for multiple comparisons between groups. Mouse survival was assessed every 12 h for 8 d after CLP. Kaplan–Meier survival curves were generated using log-rank tests. All analyses were performed using GraphPad Prism 9 (GraphPad Software, San Diego, CA, USA).

## Results

### Very high-dose and prolonged vitamin C administration enhanced survival in septic mice

To evaluate whether early, very high-dose, and/or prolonged vitamin C administration rescued mice from sepsis, experiments with varied timing, dosing, and duration of AscA administration were conducted (Fig. 1A). Specifically, AscA was intravenously injected at 1 or 6 h after CLP induction to assess whether the initial timing of vitamin C application is crucial. AscA was also administered at daily doses of 90, 180, or 360 mg/kg for 4 or 8 d to identify the optimal dose and duration of vitamin C therapy. Unexpectedly, the survival curves of mice that received AscA for 4 d were not significantly different between the 1- and 6-h groups, regardless of the dose (Fig. 1B,D). These findings were consistent when the mice received AscA for 8 d (Fig. 1C,E). In the 1-h group, the curves diverged significantly when AscA was administered at doses of 180 or 360 mg/kg/d for 8 d (Fig. 1C). However, this effect was not observed when the infusion was limited to 4 d (Fig. 1B). Similar findings were observed in the 6-h group (Fig. 1D,E). Taken together, these results indicate that a very-high dose and prolonged vitamin C infusion may increase the survival of mice with sepsis.

### Very high-dose vitamin C administration alleviated septic symptoms and reduced the inflammatory response

**Fig. 2:**
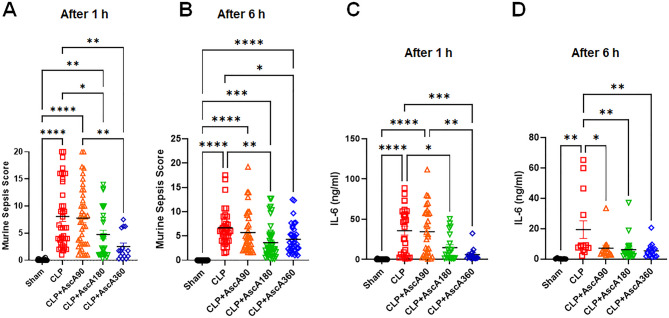
Effect of very high-dose vitamin C administration on septic symptoms and the inflammatory response. (**A**) Ascorbic acid (AscA) was injected 1 h after cecal ligation and puncture (CLP) (n = 23 in the sham; n = 41 in the CLP; n = 41 in the CLP + AscA90; n = 30 in the CLP + AscA180; and n = 15 in the CLP + AscA360). (**B**) AscA was injected 6 h after CLP (n = 21 in the sham; n = 35 in the CLP; n = 35 in the CLP + AscA90; n = 44 in the CLP + AscA180; and n = 38 in the CLP + AscA360). (**C**) AscA was injected 1 h after CLP (n = 17 in the sham; n = 27 in the CLP; n = 30 in the CLP + AscA90; n = 20 in the CLP + AscA180; and n = 15 in the CLP + AscA360). (**D**) AscA was injected 6 h after CLP (n = 7 in the sham; n = 14 in the CLP; n = 15 in the CLP + AscA90; n = 21 in the CLP + AscA180; and n = 17 in the CLP + AscA360). Data are shown as the mean ± standard error of the mean. *P < 0.05, **P < 0.01, ***P < 0.001, and ****P < 0.0001; one-way analysis of variance with Tukey’s multiple comparison test. IL: interleukin.

To determine whether septic symptoms (e.g., shortness of breath, purulent eye secretion, and low consciousness) could be alleviated by vitamin C therapy, the MSS was measured 24 h after CLP. In the 1-h group, CLP mice exhibited the highest MSS, although the score was significantly reduced when AscA was injected at doses of 180 or 360 mg/kg/d (Fig. 2A). Notably, a 90 mg/kg/d dose of the drug failed to reduce the MSS of CLP mice compared to that of mice exposed to higher AscA doses. Similar findings were observed in the 6-h group (Fig. 2B).

To further assess the impact of vitamin C on the proinflammatory response, blood was collected 24 h post-CLP, and the levels of IL-6 measured thereafter. AscA at doses of 180 or 360 mg/kg/d, but not 90 mg/kg/d, led to a significant reduction in IL-6 levels in the 1-h group (Fig. 2C). In the 6-h group, AscA at doses of 180 or 360 mg/kg/d, as well as at 90 mg/kg/d, significantly reduced IL-6 levels 24 h after CLP induction (Fig. 2D).

### Very high-dose and prolonged vitamin C administration attenuated acute lung injury in septic mice

**Fig. 3:**
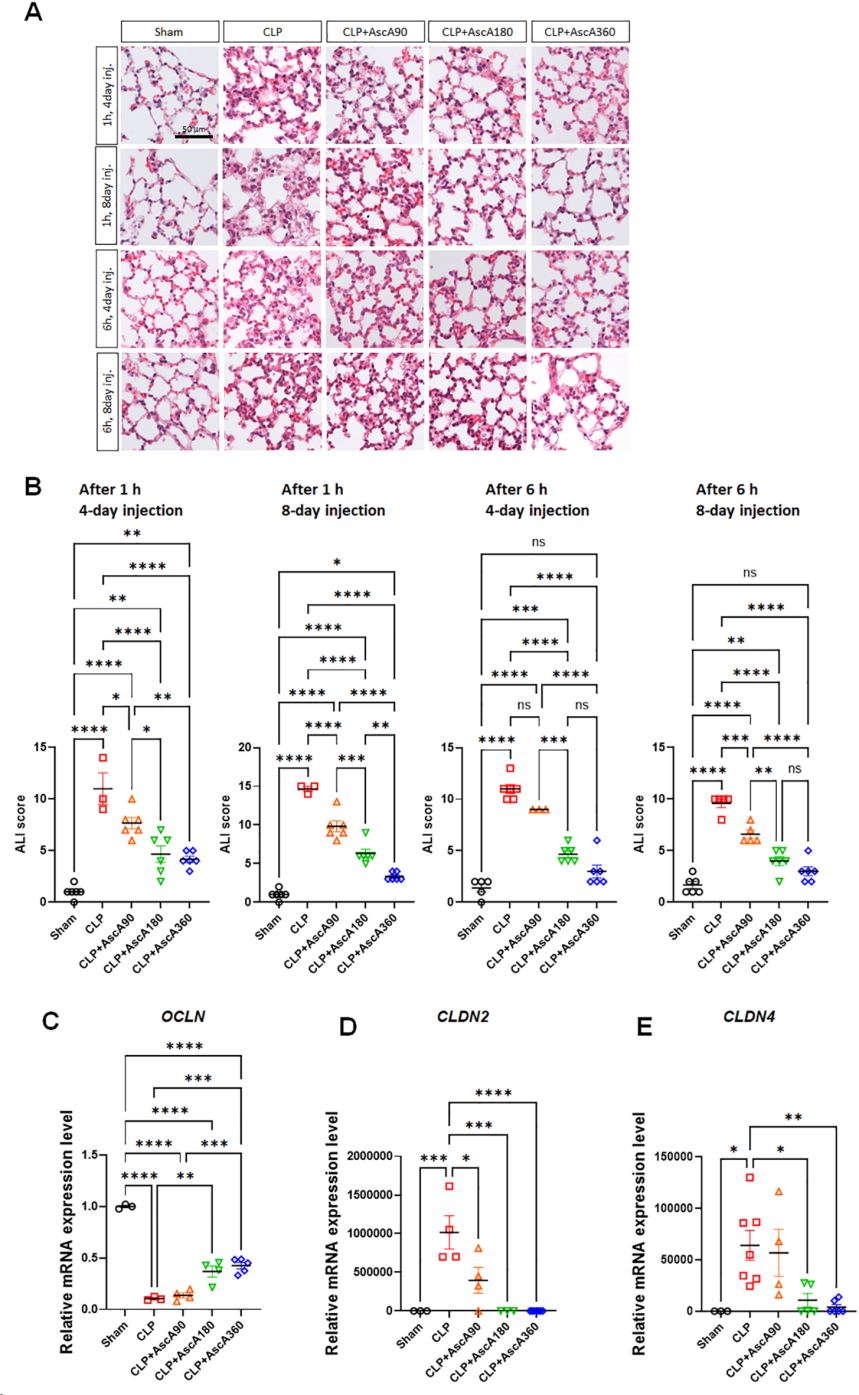
Cecal ligation and puncture (CLP) promotes acute lung injury in mice. (A) Representative hematoxylin–eosin (H&E) staining of lungs euthanized 8 d after CLP (scale bar: 50 μm). (**B**) For quantification of lung injury, a histological score was evaluated based on H&E staining (n = 3–6 per group). Quantification of (**C**) occludin (*OCLN*; n = 3–5), (**D**) claudin-2 (*CLDN2*; n = 3–7), and (**E**) claudin-4 (*CLDN4*; n = 3–7) mRNA expression levels from lungs 8 d after CLP using quantitative real-time PCR. Data are shown as the mean ± standard error of the mean. *P < 0.05, **P < 0.01, ***P < 0.001, and ****P < 0.0001; one-way analysis of variance with Tukey’s multiple comparison test. ALI: acute lung injury; AscA: ascorbic acid; inj.: injection; ns: not significant.

Inflammatory changes, such as lung edema, congestion, alveolar wall thickening, and inflammatory infiltrates, were prominent 8 d after CLP (Fig. 3A). Administration of AscA at doses of 180 or 360 mg/kg/d significantly attenuated inflammatory cell infiltration and preserved lung architecture. Notably, the degree of histological tissue injury was lowest in mice receiving AscA 1 h after CLP at a dose of 360 mg/kg/d for 8 d. Lung injury improvements were quantitatively assessed using acute lung injury (ALI) scores, which were significantly reduced after injection of AscA for 8 d, regardless of the timing or dose (Fig. 3B). In the 4-d group, AscA at doses of 180 or 360 mg/kg/d, as well as at 90 mg/kg/d, significantly reduced ALI scores when injected at 1 h after CLP induction. However, this effect was not observed when AscA was injected at 6 h after CLP induction.

Expression levels of tight junction-related protein, such as occludin (*OCLN*), claudin-2 (*CLDN2*), and claudin-4 (*CLDN4*), were evaluated using quantitative RT-PCR. CLP significantly decreased the expression of *OCLN*, whereas treatment with AscA at doses of 180 or 360 mg/kg/d for 8 d induced *OCLN* expression (Fig. 3C). Conversely, CLP induced the expression of *CLDN2* and *CLDN4*, whereas AscA treatment at doses of 180 or 360 mg/kg/d for 8 d prevented their enhanced expression (Fig. 3D,E).

### Very high-dose and prolonged vitamin C administration attenuated acute kidney injury in septic mice

**Fig. 4:**
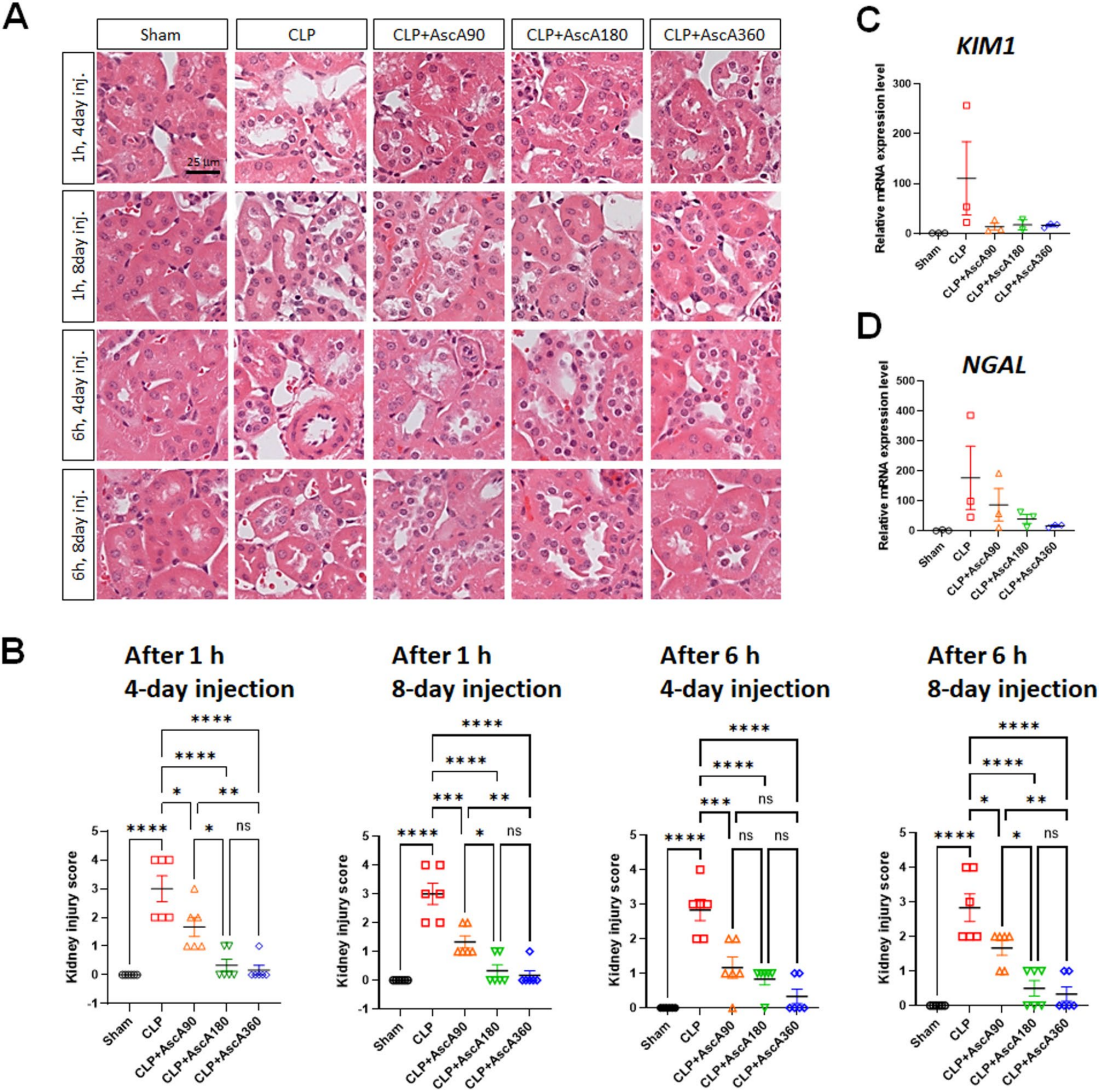
Cecal ligation and puncture (CLP) promotes acute kidney injury in mice. (**A**) Representative hematoxylin–eosin staining of kidneys euthanized 8 d after CLP (scale bar: 25 μm). (**B**) For quantification of kidney injury, a histological score was evaluated based on H&E staining (n = 6 per group). Quantification of (**C**) kidney injury molecule 1 (*KIM1*) and (**D**) neutrophil gelatinase-associated lipocalin (*NGAL*) mRNA expression levels from kidneys 8 d after CLP using quantitative real-time PCR (n = 2–3 per group). Data are shown as the mean ± standard error of the mean. *P < 0.05, **P < 0.01, ***P < 0.001, and ****P < 0.0001; one-way analysis of variance with Tukey’s multiple comparison test. AscA: ascorbic acid; inj.: injection; ns: not significant.

Kidney tissue sections from mice subjected to CLP showed severe morphological changes, including tubular dilatation and necrosis of tubular epithelial cells (Fig. 4A). However, these changes were significantly reduced by AscA application at doses of 180 or 360 mg/kg/d. Notably, the histological appearance of the kidney tissues of mice that received 360 mg/kg/d AscA for 8 d was most similar to that of the sham group. The greatest reduction in acute kidney injury (AKI) scores was achieved when AscA was infused at doses of 180 or 360 mg/kg/d for 8 d (Fig. 4B). The kidneys of mice subjected to CLP demonstrated increased expression of AKI biomarkers, including kidney injury molecule 1 (*KIM1*) and neutrophil gelatinase-associated lipocalin (*NGAL*); however, AscA treatment for 8 d, regardless of the dose, non-significantly reduced the expression of these markers (Fig. 4C,D).

### Very high-dose and prolonged vitamin C administration attenuated liver injury in septic mice

**Fig. 5:**
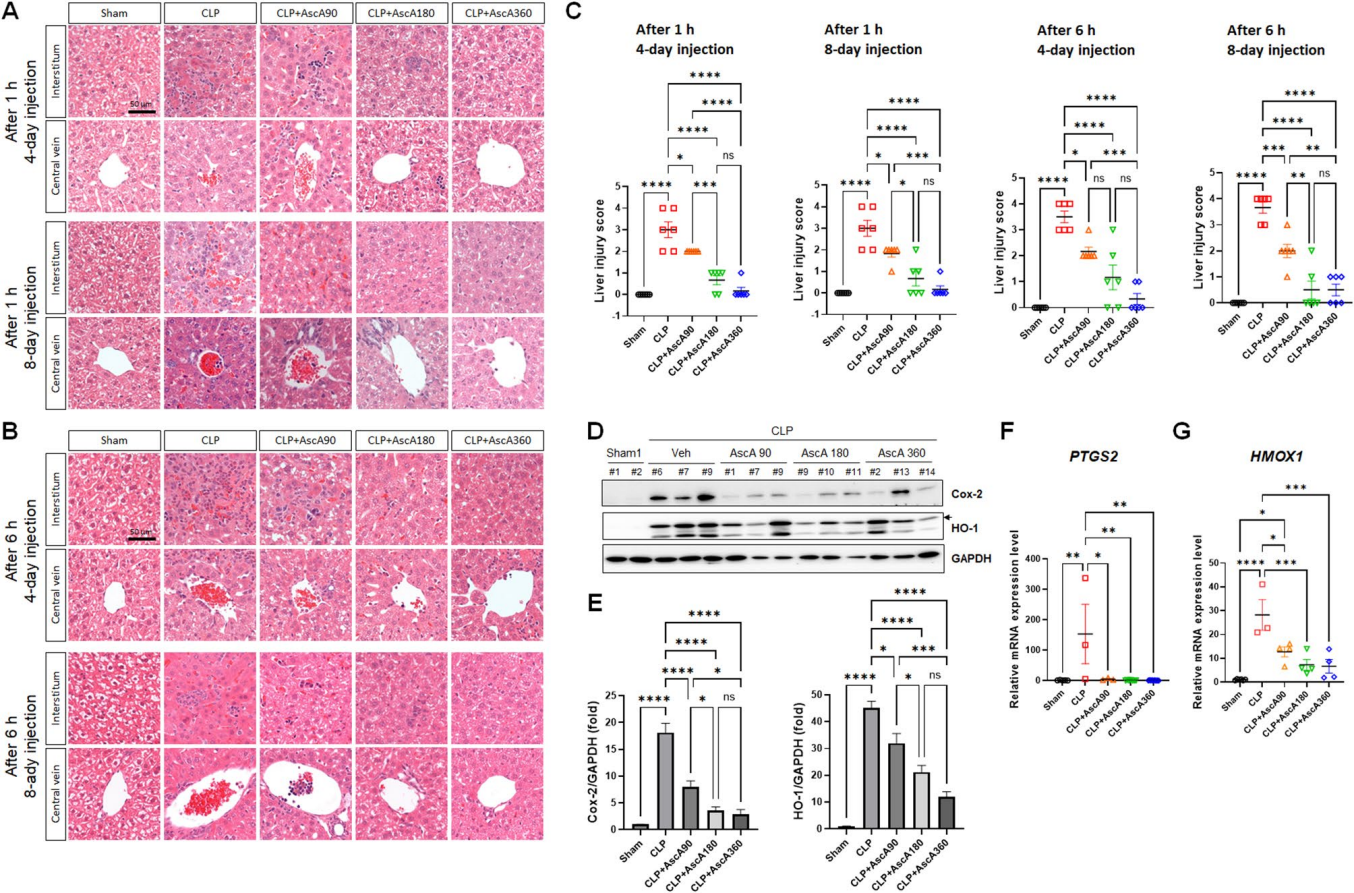
Cecal ligation and puncture (CLP) promotes liver injury in mice. (**A**,**B**) Representative hematoxylin–eosin staining of liver euthanized 8 d after CLP (scale bar: 50 μm). (**C**) For quantification of liver injury, a histological score was evaluated based on H&E staining (n = 6 per group). (**D**) Representative western blot for cyclooxygenase-2 (Cox-2) and heme oxygenase 1 (HO-1; indicated by an arrow) from liver 8 d after CLP (n = 2 in the sham; n = 3 in the CLP; n = 3 in the CLP + AscA90; n = 3 in the CLP + AscA180; and n = 3 in the CLP + AscA360). (**E**) Quantitative graphs of the protein expression levels of Cox-2 and HO-1 compared to GAPDH (n = 4). Quantification of (**F**) prostaglandin-endoperoxide synthase 2 (*PTGS2*; n = 3–9) and (**G**) heme oxygenase 1 (*HMOX1*; n = 3–6) mRNA expression levels from liver 8 d after CLP using quantitative real-time PCR. Data are shown as the mean ± standard error of the mean. *P < 0.05, **P < 0.01, ***P < 0.001, and ****P < 0.0001; one-way analysis of variance with Tukey’s multiple comparison test. AscA: ascorbic acid; ns: not significant; Veh: vehicle.

Significant morphological changes, including mononuclear cell appearance, Kupffer cell hyperplasia, and endothelialitis, were observed in the liver tissues of CLP mice (Fig. 5A,B). These were normalized in mice infused with AscA at doses of 180 or 360 mg/kg/d. Notably, AscA administration at doses of 180 or 360 mg/kg/d for 8 d significantly improved the normal hepatocyte morphology to a state similar to that observed in the sham group. Similar to AKI scores, administration of AscA at 180 or 360 mg/kg/d for 8 d resulted in the greatest reduction in acute liver injury scores (Fig. 5C). Consistent with the histological analyses, expression of the inflammatory mediators, prostaglandin-endoperoxide synthase 2 (*PTGS2*) and heme oxygenase 1 (*HMOX1*), was highly enhanced in CLP mice, whereas AscA administration for 8 d, regardless of the dose, decreased the expression of Cox-2 (encoded by *PTGS2*) and HO-1 (encoded by *HMOX1*) at both the protein and mRNA levels (Fig. 5D–G).

## Discussion

This preclinical study demonstrated that very high-dose and prolonged vitamin C injections following CLP induction in mice significantly improved survival and prevented the development of multiple organ injury. Notably, marked improvements in lung, kidney, and liver injuries and reductions in the expression of mediators of inflammation and tight junction proteins were observed. This study expands on the findings of previous reports, suggesting that the dosing and duration of vitamin C therapy play important roles in sepsis.

Sepsis is often complicated by ALI with thickened alveolar walls, loss of barrier function, and neutrophilic capillaritis^14^. Previous studies have demonstrated that vitamin C administration restores ion channel and pump expression, normalizes tight junction protein expression, and inhibits actin-cytoskeletal rearrangements^15^. In the current study, CLP induced lung histological changes consistent with those of ALI (Fig. 3A). AscA significantly reduced the levels of proinflammatory cytokines (Fig. 2C,D), attenuated lung inflammation (Fig. 3A,B), and reversed the dysregulation of tight junction protein expression (Fig. 3C–E). AKI in sepsis is characterized by microvascular dysfunction, medullary hypoperfusion, and medullary hypoxia^16,17^. Vitamin C can improve circulation from the renal cortex to the medulla and medullary nitric oxide bioavailability^18,19^. Unlike previous studies showing mild morphological damage in septic mice^20^, CLP-induced AKI presented with more severe features (Fig. 4A,B). However, infusion of AscA reduced the morphological changes and decreased the expression of *KIM1* and *NGAL* (Fig. 4C,D). In sepsis, hepatic dysfunction can be induced by vascular leakage and the migration of inflammatory cells to the sites of inflammation^20^. CLP resulted in significant liver injury (Fig. 5A–C), and AscA treatment alleviated hepatic inflammation and degradation of the extracellular matrix by attenuating the production of inflammatory mediators (Fig. 5D–G). In addition to these mechanisms, preclinical studies have indicated that vitamin C protects against sepsis-induced coagulopathy^21^.

The timing of vitamin C administration may be crucial in achieving a favorable response, although most randomized trials that failed to show a survival benefit did not assess the early and resuscitative use of vitamin C^5^. The vitamin C therapy may have been initiated after the initial cytokine storm. In murine models of sepsis, vitamin C infusion was started within 30 min of peritoneal sepsis and exhibited beneficial effects on outcomes^15,20^. Several randomized trials and retrospective analyses have suggested a reduction in mortality and faster shock reversal if treatment is initiated early^5,22,23^. However, these findings have not been uniformly reproduced. There was no significant difference in survival between the 1- and 6-h groups, regardless of the treatment duration (Fig. 1B–E). This finding is consistent with that of a recent randomized trial, in which early application of vitamin C, hydrocortisone, and thiamine did not affect mortality when compared with the results of a placebo^24^. The present results suggest that the concept of a very small therapeutic window for sepsis may not apply to vitamin C. It could be argued that the current artifactual model allows for intervention much earlier (1 h) than ever possible in the clinical setting, as patients with sepsis typically present to the emergency department with symptoms that have been present for several hours or days^25^. However, mice have different physiological responses to stimuli such as trauma or sepsis compared to humans. For instance, mice have a transient spike in cytokine production after a single dose of endotoxin, while humans have a prolonged elevation^26^. Thus, it should be noted that a 1- or 6-h interval for drug administration to mice may correspond to several hours in septic patients. Further studies are needed to explore the relationship between therapy timing and clinical outcomes.

In most trials in which no notable differences were observed between the vitamin C and placebo groups, a fixed dose of 6 g/d of the drug was infused. This dose may have been too low to affect the outcomes. Preclinical studies have shown that vitamin C at a dose of 200 mg/kg/d attenuates the proinflammatory response, improves lung injury, and prevents sepsis-induced coagulopathy^15,20^. Several meta-analyses have indicated that very high-dose (≥ 10–12 g/d) vitamin C administration is associated with decreased mortality^4,6^. Additionally, recent studies have shown that mega-doses (60–150 g/d) of vitamin C reversed renal hypoperfusion, increased urine output, and reduced vasopressor doses in a sheep model and a randomized trial of septic shock^27,28^. These findings are consistent with the results of the current study, which showed that AscA administration at doses of 180 or 360 mg/kg/d was associated with decreased mortality, disease symptoms, and organ injury, whereas AscA exposure at a dose of 90 mg/kg/d had less effect. There is a concern that vitamin C may be a pro-oxidant. However, the doses of AscA required to promote its pro-oxidant effects are reported to be much higher than those used in the present study^29^.

The effect of vitamin C may be time-dependent, with decreased mortality during the 4-d treatment period but not thereafter^30^. In previous trials that showed negative results, many patients in the intervention group did not receive vitamin C for the entire duration^31,32^. Conversely, retrospective cohort studies revealed better clinical outcomes in patients with sepsis or severe coronavirus disease 2019 who were treated with vitamin C for ≥ 5 d^33,34^. The possibility of rebound effects after abrupt cessation of vitamin C may also be a significant concern. When a high dose is discontinued abruptly, the increased metabolism in response may lead to systemic vitamin C levels that are even lower than those present before drug administration^7,35^. In a murine model of sepsis, all treated mice survived during the 4-d intervention period and only began to die after the cessation of vitamin C^8^. In the recent LOVIT trial^36^, vitamin C was administered for only 4 d, and the signal for harm might be due to rebound effects on drug cessation. The current study showed that AscA treatment at doses of 180 or 360 mg/kg/d was associated with increased survival (Fig. 1C,E) and reduced organ injuries (Figs. 3A, 4A, 5A,B) after an 8-d injection period. Moreover, AscA infusion for 4 d reduced organ injuries (Figs. 3A,B, 4A,B, 5A–C) but did not increase survival (Fig. 1B,D). These findings are consistent with those of previous studies supporting prolonged vitamin C administration.

The present study demonstrated that very high-dose and prolonged vitamin C therapy specifically protects against sepsis-induced organ injury through various mechanisms. The results emphasize that animal models can provide in-depth insights into the pathogenesis of sepsis. In contrast to clinical trials, animal studies enable the fine-tuning of a model to evaluate responses to vitamin C treatment for a given time, dose, or duration. Moreover, the experimental design enabled assessments of the efficacy of early vitamin C use based on the time of sepsis presentation. Meanwhile, mice, unlike humans, express functional L-gulono-γ-lactone oxidase and synthesize vitamin C^37^. Additionally, mice exhibit a different inflammatory system than that of humans. The murine response to endotoxin and sepsis differs in terms of tolerance and survival^38^. Translating the duration and dosage of vitamin C treatment obtained from animal models to human patients is difficult because the rates of metabolic and inflammatory responses vary across species^39^. Thus, although acceptable as proof of principle, the current results have limitations in directly applying them to clinical practice.

This study had other limitations. First, no sample size calculation was performed prior to the experiment. As we explored the efficacy of vitamin C, additional concentrations were tested. Consequently, the initial groups repeated the experiments with larger numbers of animals, while later groups used relatively smaller numbers of animals. Although sample sizes varied, statistical significance and efficacy of vitamin C remained consistent, and no samples were randomly excluded or omitted. Second, high-dose vitamin C as AscA induces metabolic acidosis^40^, which might be deleterious when treating conditions, such as sepsis with compromised buffering capacities. More frequent dosing than every 12 h with lower doses may result in rapid clearance and avoid pro-oxidant complications. Third, a recent randomized trial assessed continuous infusion of vitamin C, given that bolus injections may result in intermittent hypovitaminosis^41^. However, hypovitaminosis may still develop within several hours after discontinuing treatment with continuous infusion^7^. Thus, this limitation does not undermine the original conclusion. Fourth, a tapering dosage to avoid rebound hypovitaminosis was not employed. Fifth, vitamin C levels were not measured to assess the degree of preexisting hypovitaminosis or the extent to which the levels were increased by different doses or durations of AscA administration. Sixth, vital signs and laboratory parameters were not evaluated, although the MSS was used to assess the severity of sepsis. Finally, different clinical phenotypes of sepsis, depending on disease severity and the source of infection, were not assessed.

In conclusion, the present results support the concept that a very high-dose and prolonged vitamin C administration could be developed as a potential adjunct therapy for sepsis and septic shock. It is important to mention that improved survival would not have been observed if the use of vitamin C was limited to 4 d. The extent to which molecular events are dysregulated by the aforementioned mechanisms and normalized by vitamin C remains unclear. Further studies are warranted to determine whether this regimen is beneficial for patients with septic shock.

## Electronic supplementary material

Below is the link to the electronic supplementary material.

Supplementary Material 1

## Data Availability

All data generated or analyzed during this study are included in this published article (and its Supplementary Information files).
