# Supplementary material for: Early, very high-dose, and prolonged vitamin C administration in murine sepsis
Source: Sci Rep. 2025 May 20;15:17513. doi: 10.1038/s41598-025-02622-7 (PMC12092791; doi:10.1038/s41598-025-02622-7)

Supplementary Information

Early, very high-dose, and prolonged vitamin C administration in murine sepsis

Scientific Reports

Ok-Hyeon Kim[1], Tae Wan Kim[2], Hana Kang[3], Tae Jin Jeon[3], Eun Seo Chang[3], Hyun Jung Lee[1,3], and Won-Young Kim[2]

[1]Department of Anatomy and Cell Biology, Chung-Ang University College of Medicine, Seoul, Republic of Korea. [2]Division of Pulmonary and Critical Care Medicine, Department of Internal Medicine, Chung-Ang University Hospital, Chung-Ang University College of Medicine, Seoul, Republic of Korea. [3]Department of Global Innovative Drugs, Graduate School of Chung-Ang University, Seoul, Republic of Korea.

Supplementary Appendix. The ARRIVE Essential 10 guidelines 2.0.

Supplementary Table S1. Murine sepsis score.

Supplementary Table S2. Quantitative real-time PCR primer sequences.

Supplementary Fig. S1. Survival rate.

Supplementary Fig. S2. Time-dependent interleukin (IL)-6 level.

Supplementary Fig. S3. Full unedited gel images for Fig. 5D.

NOTE: Please save this file locally before filling in the table, DO NOT work on the file within your internet browser as changes will not be saved. Adobe Acrobat Reader (available free here) is recommended for completion.

The ARRIVE guidelines 2.0: author checklist

The ARRIVE Essential 10

These items are the basic minimum to include in a manuscript. Without this information, readers and reviewers cannot assess the reliability of the findings.

Item		Recommendation	Section/line number, or reason for not reporting
**Study design**	1	For each experiment, provide brief details of study design including:	
		a. The groups being compared, including control groups. If no control group has been used, the rationale should be stated.	p.4
		b. The experimental unit (e.g. a single animal, litter, or cage of animals).	p.4
**Sample size**	2	a. Specify the exact number of experimental units allocated to each group, and the total number in each experiment. Also indicate the total number of animals used.	p.24-26
		b. Explain how the sample size was decided. Provide details of any *a priori* sample size calculation, if done.	NA
**Inclusion and exclusion criteria**	3	a. Describe any criteria used for including and excluding animals (or experimental units) during the experiment, and data points during the analysis. Specify if these criteria were established *a priori*. If no criteria were set, state this explicitly.	p.4,5
		b. For each experimental group, report any animals, experimental units or data points not included in the analysis and explain why. If there were no exclusions, state so.	NA
		c. For each analysis, report the exact value of *n* in each experimental group.	p.24-26
**Randomisation**	4	a. State whether randomisation was used to allocate experimental units to control and treatment groups. If done, provide the method used to generate the randomisation sequence.	p.4
		b. Describe the strategy used to minimise potential confounders such as the order of treatments and measurements, or animal/cage location. If confounders were not controlled, state this explicitly.	p.4
**Blinding**	5	Describe who was aware of the group allocation at the different stages of the experiment (during the allocation, the conduct of the experiment, the outcome assessment, and the data analysis).	p.5
**Outcome measures**	6	a. Clearly define all outcome measures assessed (e.g. cell death, molecular markers, or behavioural changes).	p.5-8
		b. For hypothesis-testing studies, specify the primary outcome measure, i.e. the outcome measure that was used to determine the sample size.	p.8
**Statistical methods**	7	a. Provide details of the statistical methods used for each analysis, including software used.	p.8
		b. Describe any methods used to assess whether the data met the assumptions of the statistical approach, and what was done if the assumptions were not met.	p.8
**Experimental animals**	8	a. Provide species-appropriate details of the animals used, including species, strain and substrain, sex, age or developmental stage, and, if relevant, weight.	p.4
		b. Provide further relevant information on the provenance of animals, health/immune status, genetic modification status, genotype, and any previous procedures.	NA
**Experimental procedures**	9	For each experimental group, including controls, describe the procedures in enough detail to allow others to replicate them, including:	p.4-7
		a. What was done, how it was done and what was used.	p.4-7
		b. When and how often.	
		c. Where (including detail of any acclimatisation periods).	p.4-7
		d. Why (provide rationale for procedures).	p.4-7
**Results**	10	For each experiment conducted, including independent replications, report:	Fig. 1-5
		a. Summary/descriptive statistics for each experimental group, with a measure of variability where applicable (e.g. mean and SD, or median and range).	
		b. If applicable, the effect size with a confidence interval.	NA

Supplementary Table S1. Murine sepsis score.

Variable	Score and description
Appearance	0: Coat is smooth<br>1: Patches of hair piloerected<br>2: Majority of back is piloerected<br>3: Piloerection may or may not be present, mouse appears “puffy”<br>4: Piloerection may or may not be present, mouse appears emaciated
Level of consciousness	0: Mouse is active<br>1: Mouse is active but avoids standing upright<br>2: Mouse activity is noticeably slowed. The mouse is still ambulant<br>3: Activity is impaired. Mouse only moves when provoked, movements have a tremor<br>4: Activity severely impaired. Mouse remains stationary when provoked, with possible tremor
Activity	0: Normal amount of activity. Mouse is any of: eating, drinking, climbing, running, fighting<br>1: Slightly suppressed activity. Mouse is moving around bottom of cage<br>2: Suppressed activity. Mouse is stationary with occasional investigative movements<br>3: No activity. Mouse is stationary<br>4: No activity. Mouse experiencing tremors, particularly in the hind legs
Response to stimulus	0: Mouse responds immediately to auditory stimulus or touch<br>1: Slow or no response to auditory stimulus; strong response to touch (moves to escape)<br>2: No response to auditory stimulus; moderate response to touch (moves a few steps)<br>3: No response to auditory stimulus; mild response to touch (no locomotion)<br>4: No response to auditory stimulus. Little or no response to touch. Cannot right itself if pushed over
Eyes	0: Open<br>1: Eyes not fully open, possibly with secretions<br>2: Eyes at least half closed, possibly with secretions<br>3: Eyes half closed or more, possibly with secretions<br>4: Eyes closed or milky
Respiration rate	0: Normal, rapid mouse respiration<br>1: Slightly decreased respiration (rate not quantifiable by eye)<br>2: Moderately reduced respiration (rate at the upper range of quantifying by eye)<br>3: Severely reduced respiration (rate easily countable by eye, 0.5 s between breaths)<br>4: Extremely reduced respiration (> 1 s between breaths)
Respiration quality	0: Normal<br>1: Brief periods of labored breathing<br>2: Labored, no gasping<br>3: Labored with intermittent gasps<br>4: Gasping

Supplementary Table S2. Quantitative real-time PCR primer sequences.

Primers		Sequences (5'–3')
OCLN	Forward	CCATCTTTCTTCGGGTTTTCA
	Reverse	CTTCTGGATCTATGTACGGCTCA
CLDN2	Forward	TGAACACGGACCACTGAAAG
	Reverse	TTAGCAGGAAGCTGGGTCAG
CLDN4	Forward	TTTTGTGGTCACCGACTTTG
	Reverse	TGTAGTCCCATAGACGCCATC
KIM1	Forward	GGTCTGTATTGTTGTCGAGTGG
	Reverse	CTTCTTGGAGGACGTGTGG
NGAL	Forward	CAGCTTTACGATGTACAGCACC
	Reverse	CAAATGTTCTGATCCAGTAGCG
PTGS2	Forward	GCTTCAAACAGTTTCTCTACAACAA
	Reverse	CATTTCTTCCCCCAGCAAC
HMOX1	Forward	TGCTAGCCTGGTGCAAGATA
	Reverse	GCCAACAGGAAGCTGAGAGT
GAPDH	Forward	TGTGTCCGTCGTGGATCTGA
	Reverse	CCTGCTTCACCACCTTCTTGA

CLDN2: claudin-2; *CLDN4*: claudin-4; *HMOX1*: heme oxygenase 1; *KIM1*: kidney injury molecule 1; *NGAL*: neutrophil gelatinase-associated lipocalin; *OCLN*: occludin; *PTGS2*: prostaglandin-endoperoxide synthase 2.

Supplementary Fig. S1. Survival rate. Cecal ligation and puncture (CLP) under the conditions of 21-gauge, 1 cm ligation from the distal end of the cecum. Sham (n = 1) survived up to 72 h, and CLP mice (n = 9) had a 30% survival rate up to 72 h.

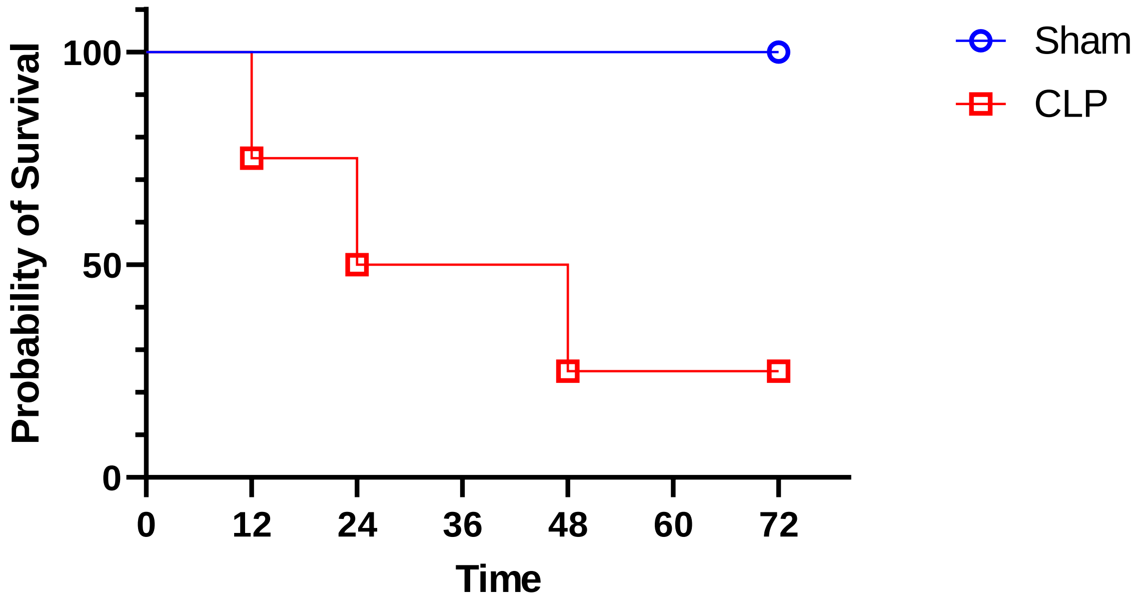

Supplementary Fig. S2. Time-dependent interleukin (IL)-6 level. Blood samples were collected from the tail at 1, 6, 12, 24, 48, and 72 h after cecal ligation and puncture (CLP) surgery, and IL-6 levels in the blood were measured using enzyme-linked immunosorbent assay. Compared with the sham model, IL-6 levels were significantly increased in the CLP model. IL-6 level measurements were stopped when CLP mice died. Conc.: concentration.

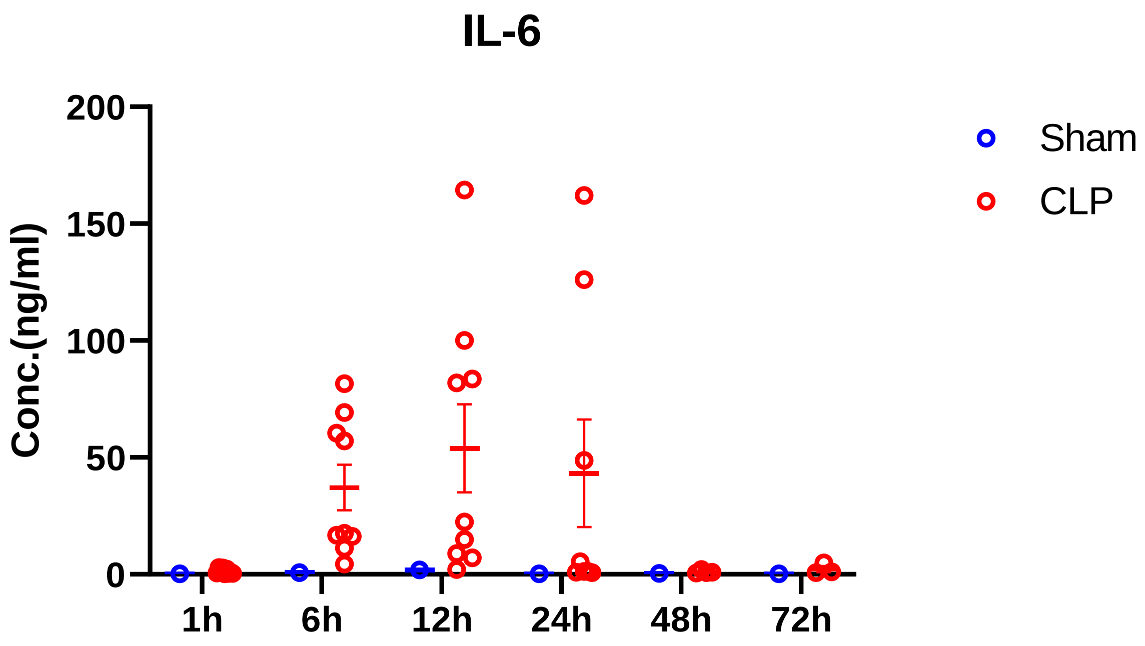

Supplementary Fig. S3. Full unedited gel images for Fig. 5D.

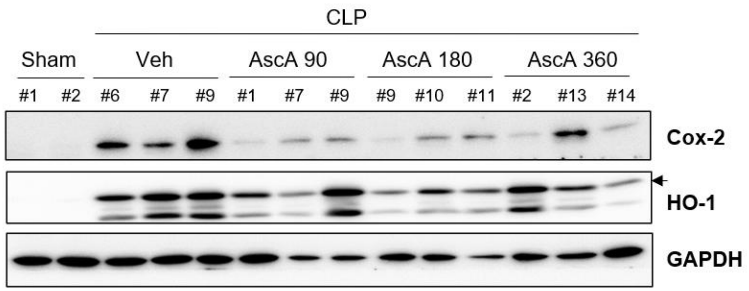

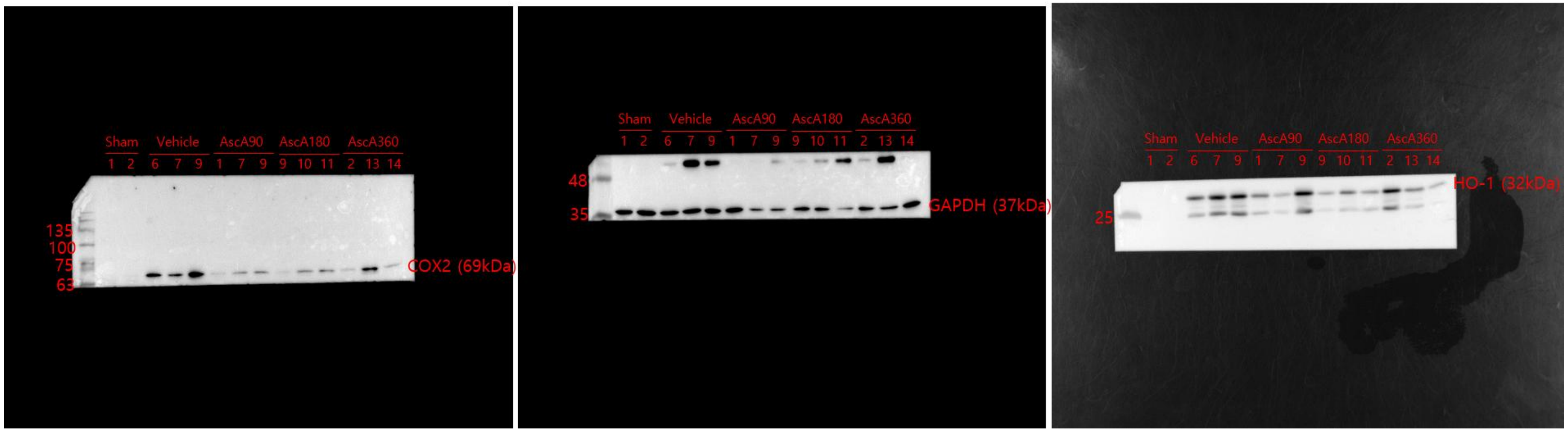

Supplement: Supplementary file 1 — Supplementary Material 1 [file 41598_2025_2622_MOESM1_ESM.pdf]
